# Association Between PET/CT Metabolic Parameters and Serum ACE and Calcium Levels in Sarcoidosis

**DOI:** 10.3390/diagnostics16020278

**Published:** 2026-01-15

**Authors:** Yaşar Incekara, Erdoğan Cetinkaya, Ramazan Eren, Reşit Akyel, Mustafa Cortuk

**Affiliations:** 1 Department of Chest Diseases, Faculty of Medicine, Karamanoğlu Mehmetbey University, 70100 Karaman, Turkey; 2 Department of Chest Diseases, Yedikule Chest Diseases and Thoracic Surgery Training and Research Hospital, University of Health Sciences, 34025 Istanbul, Turkey; erdogan.cetinkaya@sbu.edu.tr (E.C.); ramazaneren952@gmail.com (R.E.); mustafa.cortuk@sbu.edu.tr (M.C.); 3 Department of Nuclear Medicine, Yedikule Chest Diseases and Thoracic Surgery Training and Research Hospital, 34025 Istanbul, Turkey; akyelresit@hotmail.com

**Keywords:** sarcoidosis, PET/CT, angiotensin-converting enzyme (ACE), serum calcium, SUVmax, biomarkers

## Abstract

**Background:** Sarcoidosis is a multisystem inflammatory disorder characterized by non-caseating granulomas, most commonly affecting the lungs and intrathoracic lymph nodes. Angiotensin-converting enzyme (ACE) levels and calcium abnormalities are recognized biomarkers, while ^18F-fluorodeoxyglucose positron emission tomography/computed tomography (FDG-PET/CT) is increasingly used to assess disease activity. However, neither provides sufficient diagnostic accuracy alone. Therefore, this study aimed to investigate the relationship between FDG-PET/CT metabolic findings and serum ACE and calcium (Ca^2+^) levels as surrogate indicators of inflammatory metabolic intensity in sarcoidosis. **Methods:** In this retrospective single-center study, 127 patients with pulmonary sarcoidosis who underwent PET/CT at diagnosis were evaluated. Demographic and clinical data, ACE, and Ca^2+^ levels were recorded. FDG uptake in mediastinal, pulmonary, and extrapulmonary sites was analyzed, and correlations with biomarkers were assessed. **Results:** The cohort included 89 females (70.1%) and 38 males (29.9%), mean age 51.3 ± 11.9 years. FDG uptake was most frequent in mediastinal lymph nodes (84.3%) and lung parenchyma (40.9%). ACE levels correlated weakly with total SUVmax (r = 0.214, *p* = 0.019). Calcium levels correlated with extrapulmonary SUVmax (r = 0.327, *p* = 0.001) and were higher in patients with extrapulmonary involvement (*p* = 0.045). No associations were found between symptom presence and biomarkers or SUVmax values. **Conclusions:** FDG-PET/CT metabolic parameters, particularly total and extrapulmonary SUVmax, demonstrated modest yet statistically significant associations with ACE and calcium levels. These findings suggest that a combined biomarker-imaging approach may provide complementary information regarding inflammatory metabolic intensity and systemic involvement; however, the results should be interpreted as exploratory and require validation in prospective studies.

## 1. Introduction

Sarcoidosis is a multisystemic inflammatory disease characterized by the formation of non-caseating granulomas in the affected organs, with pulmonary and/or thoracic lymph node involvement in more than 90% of cases. Although its etiology has not been fully elucidated, the interaction between genetic susceptibility and environmental exposures is thought to trigger granulomatous inflammation and sustain disease activity [1,2]. The pathogenesis of sarcoidosis involves an autoimmune inflammatory process involving T cells specific to autoantigens and antibody-producing B lymphocytes. Proinflammatory cytokines released by T helper (Th) cells and macrophages play an important role in initiating the inflammatory response, resulting in the development of granuloma formation, which is the fundamental pathological feature of the disease [3,4]. Clinical presentation is highly variable, ranging from asymptomatic cases detected incidentally to severe multisystemic involvement, which has led to sarcoidosis being called “the great imitator”. Diagnosis requires careful exclusion of other causes through medical history, radiological and pathological studies, and occupational/environmental assessment [5].

Currently, no standardized diagnostic method exists for sarcoidosis. Diagnosis generally relies on three main criteria: (i) a compatible clinical presentation, (ii) characteristic radiological findings, and (iii) histological confirmation of non-necrotizing granulomatous inflammation [6]. While pulmonary and intrathoracic lymph node involvement is observed in more than 90% of patients, extrathoracic sites such as the eyes, skin, and peripheral lymph nodes are affected in 20–40% of them, and clinically significant involvement of the central nervous system, heart, liver, spleen, bone, or kidneys occurs less frequently [7].

Although laboratory parameters in sarcoidosis are nonspecific, several biomarkers provide supportive diagnostic value. Serum angiotensin-converting enzyme (ACE) levels, secreted by activated mononuclear phagocytes, are elevated in approximately 50% of patients [8]. In addition, hypercalcaemia may occur due to excessive extrarenal production of 1,25-dihydroxyvitamin D by activated macrophages, which increases intestinal calcium absorption and may lead to complications such as hypercalciuria and nephrolithiasis [9]. Previous studies have reported hypercalcaemia in 3–12% of patients; in one large retrospective series, it was observed in 6% of 1606 sarcoidosis cases [10].

Radiological imaging plays a central role in diagnosis. While conventional chest radiography and computed thorax tomography (CTT) remain standard tools, they may be limited in assessing the inflammatory burden and predicting prognosis. In recent years, ^18F-fluoro-2-deoxy-D-glucose positron emission tomography/computed tomography (^18F-FDG PET/CT) has gained importance in sarcoidosis because of its ability to detect increased glucose metabolism in activated neutrophils, macrophages, and lymphocytes, thus reflecting active inflammation [11]. Several studies have suggested that PET/CT may provide valuable information for determining disease severity and guiding treatment decisions [12]. Despite these advances, both ACE levels and PET/CT have limitations when used alone. ACE lacks sufficient sensitivity and specificity, while PET/CT, although highly sensitive, may yield nonspecific uptake in other granulomatous or inflammatory conditions. Therefore, there is a need for studies evaluating whether combining these modalities can enhance diagnostic accuracy. In this study, we aimed to investigate the correlations between PET/CT findings and ACE and calcium levels in patients with sarcoidosis, as well as to evaluate their potential value as complementary tools in reflecting inflammatory intensity rather than simply improving diagnostic accuracy.

## 2. Materials and Methods

### 2.1. Study Design and Population

This retrospective observational study was conducted at Yedikule Training and Research Hospital. Between 1 January 2020 and 30 September 2024, the medical records of 210 adult patients (>18 years) diagnosed with pulmonary sarcoidosis through systematic evaluation, including histological confirmation by endobronchial ultrasound-guided transbronchial needle aspiration (EBUS-TBNA), were reviewed. Patients without pulmonary function tests (PFTs) or PET/CT imaging at the time of diagnosis, or with comorbidities that could affect inflammatory markers (such as autoimmune diseases or active malignancy), were excluded. Patients receiving medications affecting the renin–angiotensin–aldosterone system (ACE inhibitors or angiotensin II receptor blockers) were also excluded. After applying these criteria, 127 patients were included in the final analysis.

### 2.2. Data Collection

Demographic characteristics, clinical features (disease duration, radiological stage, and treatments administered), laboratory results (serum ACE levels, serum biochemical parameters, and complete blood count), and pulmonary function test values were obtained from the electronic medical record system. None of the patients were receiving drugs known to interfere with ACE activity. 18F-FDG PET/CT images were retrieved from the institution’s data repository and retrospectively reviewed using the same image review workstation (Advantage Workstation 4.7, GE Healthcare, Chicago, IL, USA). The maximum standardized uptake value (SUVmax) was measured for mediastinal, pulmonary, and extrapulmonary lesions using a three-dimensional region of interest (ROI). In cases where more than one area of FDG uptake was detected (i.e., pulmonary plus extrapulmonary, lymph node plus pulmonary, lymph node plus extrapulmonary, or all three areas), total SUVmax values were calculated by adding the SUVmax values of each region. In cases where more than one FDG-positive lymph node or lesion was present within an area, the lesion with the highest SUVmax was selected. No background correction and/or normalization was applied to raw SUVmax values. We aimed to assess inflammatory metabolic intensity using SUVmax, which represents the highest focal FDG uptake within metabolically active lesions. As TLG and MTV are not routinely assessed, we considered SUVmax measurement, which reflects inflammatory metabolic intensity, to be a practical and widely used parameter in routine PET/CT assessment rather than a measure of total granuloma burden.

### 2.3. PET/CT Protocol

All patients underwent PET/CT scanning after at least six hours of fasting, provided that fasting blood glucose levels were <150 mg/dL. Imaging was performed using a dedicated PET/CT scanner (Discovery 600, GE Medical Systems, Milwaukee, WI, USA) approximately 60 min after intravenous administration of 3.7 MBq/kg (~0.1 mCi/kg) of ^18F-FDG. A low-dose non-contrast CT scan was acquired (tube voltage: 120 kV; tube current: 100–210 mAs), followed by a three-dimensional PET acquisition from the skull base to mid-thigh with an acquisition time of 1.75 min per bed position. Semi-quantitative analysis was performed by calculating SUVmax for mediastinal and abdominal lymph nodes, as well as pulmonary lesions. SUVmax is known to be scanner-dependent and noise-sensitive; therefore results should be interpreted cautiously.

### 2.4. Statistical Analysis

Statistical analyses were performed using SPSS software, version 26.0 (IBM Corp., Armonk, NY, USA). Continuous variables were expressed as mean ± standard deviation or median (minimum–maximum), and categorical variables as frequencies and percentages. The normality of distribution was assessed using the Shapiro–Wilk test and visual inspection of histograms and box plots. For group comparisons, Student’s *t*-test was used for normally distributed variables, and the Mann–Whitney U test for non-normally distributed variables. Categorical variables were compared using the Chi-square test or Fisher’s exact test, where appropriate. Correlations between continuous variables were evaluated using Spearman’s rank correlation coefficient. A two-tailed *p*-value < 0.05 was considered statistically significant, and results were reported with 95% confidence intervals (Table 1).

## 3. Results

The study was conducted with a total of 127 patients, of whom *n* = 89 (70.1%) were female and *n* = 38 (29.9%) were male. The ages of the patients ranged between 27 and 82 years and the mean age was 51.35 ± 11.95 years.

Comorbidity was observed in 54.3% (*n* = 69) of the participants. The most common comorbidity was DM in 24.6% (*n* = 17).

Symptoms were present in 66.9% (*n* = 85) of the cases. The most common symptoms were shortness of breath (36.5%) and cough (40%). Demographic characteristics, symptoms and comorbidities of the patients are given in Table 2.

When the radiological stages were examined, it was found that 2.4% (*n* = 3) of the patients were stage 0, 51.2% (*n* = 65) were stage 1, 45.2% (*n* = 54) were stage 2, 3.1% (*n* = 4) were stage 3, and 0.8% (*n* = 1) were stage 4.

PET CT was performed in all participants (*n* = 127). When FDG uptake was analyzed, the most common site of uptake was the mediastinal lymph nodes with 84.3% and lung parenchyma with 40.9%. 13.4% (*n* = 17) had no FDG uptake (Table 3).

ACE measurements of the participants ranged between 3 and 191 ıu/mL, with a mean value of 58.61 ± 35.91 U/L.

Serum calcium values of the participants ranged between 7.9 and 14, and the mean value was 9.62 ± 0.67 mg/dL.

**Figure 1:**
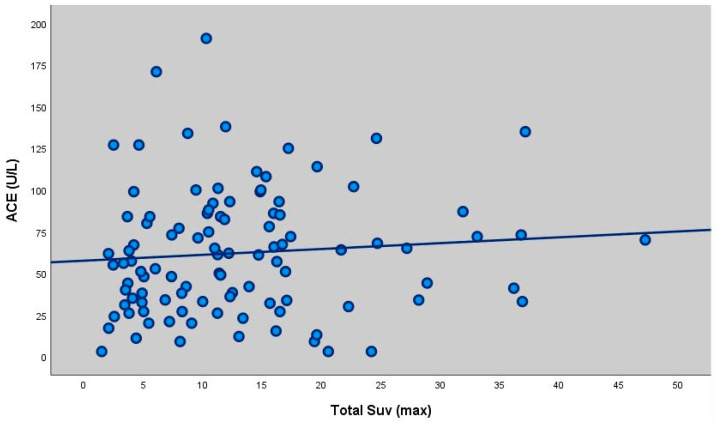
Scatter plot showing the correlation between ACE levels and total SUVmax (r = 0.214, *p* = 0.019). There was no statistically significant correlation between ACE measurements and LAP SUV (max) and Parenchymal SUV (max) measurements of the participants (*p* > 0.05). There was no statistically significant correlation between extrapulmonary SUVmax values and ACE levels of the participants (*p* > 0.05). There was no statistically significant correlation between calcium levels and Total SUV (max), LAP SUV (max) and Parenchymal SUV (max) measurements of the participants (*p* > 0.05). There was a statistically significant weak positive correlation between extrapulmonary FDG measurements and calcium values (extrapulmonary FDG value increased as calcium value increased) (r = 0.327; *p* = 0.001; *p* < 0.01).

There was a statistically significant weak positive correlation between ACE measurements and Total SUV (max) measurements (as ACE measurement increased, Total SUV value increased) (r = 0.214; *p* = 0.019; *p* < 0.05) (Table 4, Figure 1).

Accompanying disease, presence of symptoms, smoking status, age and gender did not show statistically significant differences in LAP SUV (max), total SUV (max), parenchymal SUV (max) and extrapulmonary SUV (max) values (all *p* > 0.05).

No statistically significant difference was found between the ACE measurements of the patients according to the presence of extrapulmonary involvement (*p* < 0.05) (Table 5).

**Figure 2:**
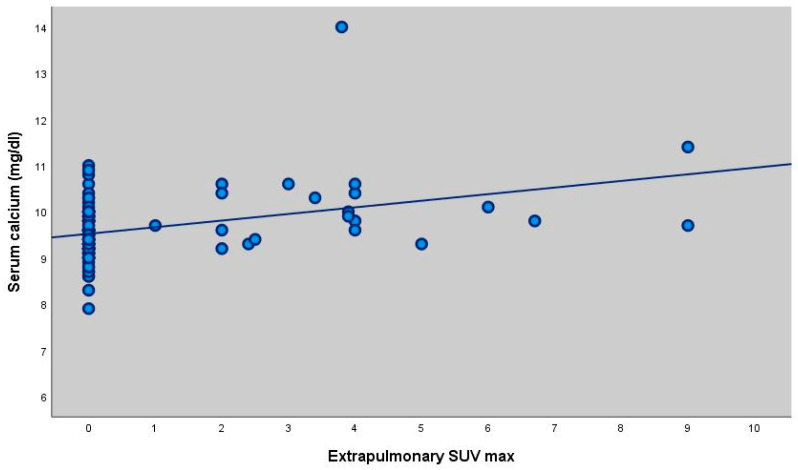
Scatter plot showing the correlation between serum calcium levels and extrapulmonary SUVmax (r = 0.327, *p* = 0.001).

**Figure 3:**
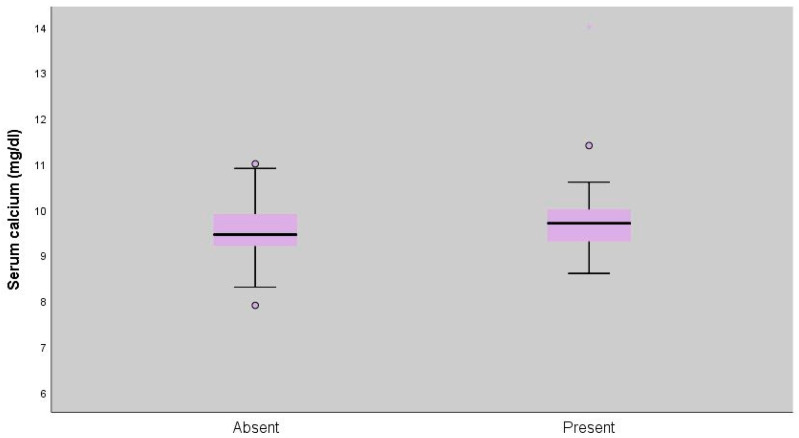
Distribution of serum calcium levels according to extrapulmonary involvement (*p* = 0.045).

Serum calcium measurements of patients with extrapulmonary involvement were statistically significantly higher (*p* = 0.045; *p* < 0.05) (Table 5, Figure 2 and Figure 3).

Table 6 shows that there was no statistically significant difference between ACE, calcium and total SUV max measurements of the patients according to the presence of symptoms (*p* < 0.05).

## 4. Discussion

In this study, we investigated the relationship between FDG-PET/CT metabolic parameters and serum ACE and calcium levels as surrogate indicators of inflammatory metabolic intensity in patients with biopsy-proven sarcoidosis. The main findings were a modest but statistically significant correlation between total SUVmax and ACE levels, and a stronger association between extrapulmonary metabolic involvement and serum calcium levels. In contrast, no significant associations were observed between PET/CT findings, biochemical markers, and the presence of clinical symptoms. These results suggest that PET/CT-derived metabolic intensity and conventional serum biomarkers may reflect different and complementary aspects of the inflammatory process in sarcoidosis, rather than directly indicating clinical disease severity or diagnostic performance.

Epidemiological studies have reported that the incidence of sarcoidosis may be higher in women than in men, although some findings are conflicting [14]. In our cohort, female predominance was evident (70.1%), which is consistent with most of the literature.

18F-fluoro-2-deoxyglucose positron emission tomography combined with computed tomography (18F-FDG PET/CT) is a highly sensitive imaging modality capable of identifying sites of active inflammation, often before morphological changes are visible on conventional imaging [15]. Several studies have demonstrated its usefulness in reliably diagnosing suspected pulmonary sarcoidosis [16]. Furthermore, whole-body FDG- PET has been shown to reflect metabolically active granulomatous involvement in patients with biopsy-proven sarcoidosis [15].

Since the 1930s, sarcoidosis has been recognized as a cause of hypercalcaemia [17]. Sarcoidosis-related hypercalcaemia is clinically important because it may result in serious morbidity and often requires long-term treatment. Identifying patients at higher risk could enable earlier intervention and closer follow-up [18]. In our study, no significant correlation was found between SUVmax values and serum calcium levels. However, calcium levels were significantly higher in patients with extrapulmonary involvement. This finding suggests that extrapulmonary disease may be a risk factor for hypercalcaemia, underlining the need for closer monitoring in such cases. Although serum calcium levels were statistically higher in patients with extrapulmonary involvement, the effect size was small to moderate (Cohen’s d = 0.39). This finding suggests that while extrapulmonary metabolic involvement may be associated with calcium dysregulation, the magnitude of this difference is modest and may not translate into a strong clinical discriminator at the individual patient level. Therefore, this association should be interpreted as biologically relevant but clinically limited.

Serum ACE levels are elevated in many patients with sarcoidosis and were initially thought to parallel disease activity [19]. Despite its proposed role in disease monitoring, the utility of ACE as a reliable biomarker remains controversial due to inconsistent findings across studies [20]. In our study, the mean ACE value was above the reference range, in line with reports suggesting frequent ACE elevation in sarcoidosis.

When ACE levels were compared with PET/CT results, no significant correlation was found with parenchymal or lymph node SUVmax values. However, a weak but significant positive correlation was observed between total SUVmax and ACE levels (*p* = 0.019), suggesting that higher ACE levels may be associated with greater overall inflammatory metabolic intensity. This finding contrasts with previous reports [12,21] that reported no association between SUVmax values and biochemical markers of disease activity.

Another notable result was the absence of significant differences in SUVmax, ACE, or calcium levels between symptomatic and asymptomatic patients. This finding highlights that elevations in biochemical markers and PET/CT-derived metabolic activity may not always correlate with clinical presentation. The absence of statistically significant differences between symptomatic and asymptomatic patients was accompanied by very small effect sizes across ACE, calcium, and total SUVmax measurements, indicating a negligible association between clinical symptom burden and both biochemical markers and PET-derived metabolic intensity.

In conclusion, our study demonstrated a significant correlation between total SUVmax and ACE levels, as well as higher calcium levels in patients with extrapulmonary involvement. These results suggest that combined evaluation of PET/CT findings and biochemical markers such as ACE and calcium may provide additional insights into inflammatory metabolic intensity and systemic involvement in sarcoidosis.

### Strengths and Limitations

The main strength of our study is that it evaluates both PET/CT parameters and biochemical markers (ACE and calcium) together in a relatively large cohort of patients with biopsy-proven sarcoidosis. The combined analysis of imaging and laboratory data provides a more comprehensive assessment of disease activity and systemic involvement. Additionally, all patients underwent PET/CT with a standardized imaging protocol, which increases the reliability of the metabolic measurements.

However, this study also has limitations. Its retrospective and single-center design may limit the generalizability of the findings. The cross-sectional nature of the data does not allow for longitudinal evaluation of changes in biomarkers or PET/CT parameters during follow-up. Furthermore, although serum ACE and calcium levels were evaluated, other potentially relevant biomarkers of inflammation were not included, which may have provided additional insight. Finally, the absence of histopathological correlation with PET/CT findings for all extrapulmonary sites is another limitation.

## 5. Conclusions

In this study, we demonstrated that serum ACE levels showed a positive correlation with total SUVmax values obtained from PET/CT, while calcium levels were higher in patients with extrapulmonary involvement. These findings suggest that PET/CT metabolic parameters and biochemical markers such as ACE and calcium may provide complementary information regarding inflammatory metabolic intensity and systemic involvement in sarcoidosis. However, the results should be considered exploratory and hypothesis-generating, and require confirmation in prospective multicenter studies before any clinical application can be considered.

## Figures and Tables

**Table 1:** Interpretation of Cohen’s Effect Size [13].

Cohen’s d	Interpretation
0.20	Small effect
0.50	Medium effect
0.80	Large effect

Cohen, J. (1988). Statistical Power Analysis for the Behavioral Sciences (2nd ed.).

**Table 2:** Distribution of Descriptive Characteristics.

		*n* (%)
**Gender**	Female	89 (70.1)
	Male	38 (29.9)
**Age**	Mean ± Ss	51.35 ± 11.95
**Presence of comorbidity**	None	58 (45.7)
	Present	69 (54.3)
**° Additional disease (*n* = 69)**	COPD, Asthma	6 (8.7)
	HT	16 (23.2)
	Previous malignancy	6 (8.7)
	HF-AF	11 (15.9)
	DM	17 (24.6)
	Late TB	3 (4.3)
	Other	11 (15.9)
**Smoking**	Never smoker	22 (17.32)
	Current smoker Former smoker	16 (12.60) 89 (70.08)
**Presence of Symptom**	No	42 (33.1)
	Yes	85 (66.9)
**° Symptoms (*n* = 85)**	Shortness of breath	31 (36.5)
	Cough	34 (40.0)
	Chest pain	3 (3.5)
	Itching, swelling	1 (1.2)
	Fatigue, loss of appetite, joint pain	16 (18.8)
**Staging**	Stage 0	3 (2.4)
	Stage 1	65 (51.2)
	Stage 2	54 (45.2)
	Stage 3	4 (3.1)
	Stage 4	1 (0.8)

° More than one option is selected. (COPD: Chronic Obstructive Pulmonary Disease, HT: Hypertension, TB: Tuberculosis, DM: Diabetes Mellitus).

**Table 3:** Distribution of PET-CT, ACE, Calcium Findings.

		*n* (%)
**° PET CT Involvement**	Mediastinal lymph nodes	107 (84.3)
	Lung parenchyma	52 (40.9)
	Heart	3 (2.4)
	Spleen	8 (6.3)
	Musculoskeletal Thyroid	13 (10.2) 2 (1.6)
	Skin	1 (0.8)
	Peripheral lap	24 (18.9)
	Liver/Stomach/GI	9 (7.1)
	Endometrium Vagina Prostate Ovary Rectum	2 (1.6) 1 (0.8) 1 (0.8) 2 (1.6) 1 (0.8)
	No involvement	17 (13.4)
**LAM SUV (max)**	Mean ± Ss	8.23 ± 5.09
	Median (Min-Max)	6.8 (2–23.1)
**Total SUV (max)**	Mean ± Ss	11.34 ± 9.16
	Median (Min-Max)	11.3 (1.5–47.2)
**Parenchymal SUV (max)**	Mean ± Ss	4.80 ± 3.08
	Median (Min-Max)	3.5 (1.2–13.5)
**ACE (U/L)**	Mean ± Ss	58.61 ± 35.91 (0–52)
	Median (Min-Max)	51 (3–191)
**Serum calcium (mg/dL)**	Mean ± Ss	9.62 ± 0.67 (8–10.6)
	Median (Min-Max)	9.6 (7.9–14)

° More than one option is selected. (PET CT: Positron Emission Tomography (PET) and Computed Tomography (CT), GI: Gastrointestinal, SUV(max): Maximum Standard Unit Value, ACE: angiotensin converting enzyme).

**Table 4:** Correlation between ACE, serum calcium levels and SUVmax values.

	ACE (U/L)		Calcium (mg/dL)	
	r	*p*	r	*p*
**LAM SUV (max)**	0.107	0.310	−0.037	0.725
**Total SUV (max)**	0.214	0.019 *	0.147	0.137
**Parenchymal SUV (max)**	−0.057	0.691	0.050	0.724
**Extrapulmonary SUV (max)**	0.173	0.052	0.327	0.001 **

r: Spearman Correlation Coefficient * *p* < 0.05, ** *p* < 0.01.

**Table 5:** Comparison of ACE and serum calcium levels according to extrapulmonary involvement.

		Extrapulmonary Involvement		*p*	
		None (*n* = 91)	There Is (*n* = 35)		Effect Size (d)
**ACE**	Mean ± Ss	56.14 ± 36.6	65.04 ± 33.71	^a^ 0.214	0.248
	Median (Min-Max)	49 (3–191)	68 (12–135)		
**Serum Calcium**	Mean ± Ss	9.54 ± 0.53	9.81 ± 0.92	^a^ 0.045 *	0.388
	Median (Min-Max)	9.5 (7.9–11)	9.7 (8.6–14)		

^a^ Student *t* test; d: Cohen’s d. * *p* < 0.05.

**Table 6:** Comparison of ACE, calcium, and total SUVmax according to the presence of symptoms.

		Presence of Symptoms		*p*	
		None (*n* = 85)	Yes (*n* = 42)		Effect Size (d)
**ACE**	Mean ± Ss	56.47 ± 31.1	59.64 ± 38.13	^a^ 0.985	0.088
	Median (Min-Max)	57.3 (3–127)	51 (3–191)		
**Serum Calcium**	Mean ± Ss	9.53 ± 0.55	9.65 ± 0.72	^a^ 0.360	0.173
	Median (Min-Max)	9.5 (7.9–10.8)	9.6 (8.3–14)		
**Total SUV (max)**	Mean ± Ss	12.71 ± 8.54	13.21 ± 9.49	^b^ 0.969	0.054
	Median (Min-Max)	11.3 (1.5–36.2)	11.4 (2.1–47.2)		

^a^ Student’s *t* test; ^b^ Mann–Whitney U test; d: Cohen’s d.

## Data Availability

The raw data supporting the conclusions of this article will be made available by the authors on request.
